# TMT-Based Quantitative Proteomics Analysis of Synovial Fluid-Derived Exosomes in Inflammatory Arthritis

**DOI:** 10.3389/fimmu.2022.800902

**Published:** 2022-03-11

**Authors:** Yukai Huang, Yuqi Liu, Qidang Huang, Shanmiao Sun, Zhuyi Ji, Lixin Huang, Zhi Li, Xuechan Huang, Weiming Deng, Tianwang Li

**Affiliations:** ^1^ Department of Rheumatology and Immunology, Guangdong Second Provincial General Hospital, Guangzhou, China; ^2^ Guangdong Second Provincial General Hospital, University of South China, Hengyang, China; ^3^ Department of Rheumatology and Immunology, Zhaoqing Central People’s Hospital, Zhaoqing, China

**Keywords:** inflammatory arthritis, synovial fluid, exosomes, proteomics analysis, biomarkers

## Abstract

**Objectives:**

To compare the proteomics of synovial fluid (SF)-derived exosomes in rheumatoid arthritis (RA), axial spondyloarthritis (axSpA), gout, and osteoarthritis (OA) patients.

**Methods:**

Exosomes were separated from SF by the Exoquick kit combined ultracentrifugation method. Tandem mass tags (TMT)-labeled liquid chromatography mass spectrometry (LC-MS/MS) technology was used to analyze the proteomics of SF-derived exosomes. Volcano plot, hierarchical cluster, gene ontology (GO), and Kyoto Encyclopedia of Genes and Genomes (KEGG) pathway analysis were conducted.

**Results:**

A total of 1,678 credible proteins were detected. Sixty-nine differentially expressed proteins were found in gout, compared with OA, axSpA, and RA simultaneously. Twenty-five proteins were found highly expressed in gout uniquely, lysozyme C and protein S100-A9 included, whose bioinformatic analysis was significantly involved in “neutrophil degranulation” and “prion diseases”. Eighty-four differentially expressed proteins were found in axSpA, compared with OA, gout, and RA simultaneously. Thirty-nine proteins were found highly expressed in axSpA uniquely, RNA-binding protein 8A and protein transport protein Sec24C included, whose bioinformatic analysis was significantly involved in “acute-phase response” and “citrate cycle”. One hundred and eighty-four differentially expressed proteins were found in RA, compared with OA, gout, and axSpA simultaneously. Twenty-eight proteins were found highly expressed in RA uniquely, pregnancy zone protein (PZP) and stromelysin-1 included, whose bioinformatic analysis was significantly involved in “serine-type endopeptidase inhibitor activity” and “complement and coagulation cascades”. Enzyme-linked immunosorbent assay (ELISA) result showed that the exosome-derived PZP level of SF in RA was higher than that in OA (*p* < 0.05).

**Conclusion:**

Our study for the first time described the protein profiles of SF-derived exosomes in RA, axSpA, gout, and OA patients. Some potential biomarkers and hypothetical molecular mechanisms were proposed, which may provide helpful diagnostic and therapeutic insights for inflammatory arthritis (IA).

## 1 Introduction

Inflammatory arthritis (IA) is characterized by synovial inflammation and synovial hyperplasia leading to joint damage, and mainly includes gout, axial spondyloarthritis (axSpA), and rheumatoid arthritis (RA) (1, 2). The incidence and prevalence of IA are increasing in recent years, bringing a great burden to the society and family (3). IA is a multifactorial disease driven by the complex interplay of genetic predisposition and environmental risk factors (4, 5). It is of great importance to explore the pathogenesis and distinguishing biomarkers of IA.

Synovial fluid (SF) provides a low-friction environment that allows joint movement and nourishes surrounding tissues with nutrition (6). When patients suffer from IA, the volume and composition of SF would change. Immune cells, such as macrophages, lymphocytes, and neutrophils in the SF, would be activated and produce inflammatory cytokines and proteolytic enzymes, playing an important role in immune responses and bone destruction (7, 8). Exosomes are small particles with a diameter of 30–120 nm, which contain proteins and RNAs and secreted by various cell types (9). Exosomes in SF have a close relationship with the pathogenesis of arthritis, which can lead to inflammation, degeneration of cartilage, and destruction of joints (10). Song (11) compared the role of SF-derived exosomes from IA in osteoclast differentiation and found that SF-derived exosomes of RA patients may contain the disease-specific “synovial signature of osteoclastogenesis”. Recent studies showed that SF-derived exosomes can be the biomarker for different stages of joint disease (12). Foers (13) explored the proteomics of extracellular vesicles (EVs) in RA patients and indicated that 45 and 135 EV-associated proteins were significantly elevated in RA with high-level inflammation than in RA with low-level inflammation and osteoarthritis (OA), respectively. However, no study has compared the proteomics of SF-derived exosomes in RA, axSpA, gout, and OA patients simultaneously.

In the present study, SF-derived exosomes were isolated from RA, axSpA, gout, and OA patients. The Tandem Mass Tags (TMT)-labeled quantitative proteomics technique was used to explore the protein profiles of exosomes. The data may advance current insights into the molecular mechanisms of IA and serve as biomarkers for IA.

## 2 Materials and Methods

### 2.1 Participants

A total of 42 gout, 30 RA, 10 axSpA, and 18 OA patients in our hospital from December 2018 to October 2020 were enrolled in the study. The inclusion criteria were as follows: patients with pain in the knee joints, and SF was collected by arthrocentesis. Gout was diagnosed on the ACR/EULAR 2015 criteria. RA was diagnosed on the ACR/EULAR 2010 criteria. AxSpA was diagnosed on the 2009 ASAS classification criteria. OA was diagnosed on x-ray findings of reduced medial joint space. SF was centrifuged and the supernatant was stored at −80°C. The study was approved by the EC office of the Guangdong Second Provincial General Hospital (2017-FSMY-009).

### 2.2 Isolation and Characterization of Exosomes

Exosomes were isolated by ExoQuick™ kit (System Biosciences) combined ultracentrifugation method. The size and concentrations of exosomes were detected by high sensitivity flow cytometry (HSFC) for nanoparticle analysis. Western blot was used to examine the level of exosomes markers (TSG101 and CD81).

### 2.3 Protein Digestion

SF-derived exosomes from 9 gout, 9 RA, 9 axSpA, and 9 OA patients were selected randomly. Exosomes of every 3 patients in the same group were mixed as one sample to conduct protein digestion. Corresponding volume of 25 mM DL-dithiothreitol was added to each sample. The iodoacetamide was added. Then, 6 times of the volume of precooled acetone was added to precipitate the protein. After precipitation, the sample was centrifuged for collecting the precipitate. Enzymolysis diluent [protein:enzyme = 50:1 (m/m)] was used to redissolve the protein precipitate, followed by lyophilization.

### 2.4 TMT Labeling

The lyophilized samples were resuspended in 100 mM tetraethylammonium bromide and transferred into new tubes. Acetonitrile was added to TMT reagent. Then, 10 μl of the TMT label reagent was added to each sample and incubated for 1 h. Finally, hydroxylamine was added to each sample and incubated for 15 min to terminate reaction. The labeling peptide solutions were lyophilized.

### 2.5 RPLC Analysis

Reversed-phase (RP) separation was performed on an 1100 HPLC System (Agilent) with an Agilent Zorbax Extend RP column (5 μm, 150 mm × 2.1 mm). RP gradient was mobile phases A (2% acetonitrile in HPLC water) and B (90% acetonitrile in HPLC water). Samples were collected for 8-60 min, and eluent was collected in centrifugal tubes 1-15 every minute in turn. Samples were recycled in this order until the end of gradient. The separated peptides were lyophilized for mass spectrometry.

### 2.6 Mass Spectrometry Analysis

All analyses were performed by a Q-Exactive mass spectrometer (Thermo, USA) equipped with a Nanospray Flex source (Thermo, USA). Samples were loaded and separated by a C18 column (15 cm × 75 μm) on an EASY-nLCTM 1200 system (Thermo, USA). Full MS scans were acquired in the mass range of 350-1650 m/z with a mass resolution of 120,000 with an AGC target of 3e6. MS/MS spectra were obtained with a resolution of 60,000 with an AGC target of 1e5 and a max injection time of 120 ms. The Q-E dynamic exclusion was set for 40.0 s and run under positive mode. Proteome Discoverer (v.2.4) was used to search all of the Q Exactive raw data. Database search was performed with trypsin digestion specificity. A global false discovery rate (FDR) was set to 0.01 and protein groups considered for quantification required at least 2 peptides.

### 2.7 ELISA

SF-derived exosomes from 7 gout, 7 RA, 8 axSpA, and 8 OA patients were selected randomly for ELISA. The ELISA Kits (CSB-EL019131HU, CUSABIO BIOTECH) were performed to detect the pregnancy zone protein (PZP) level of SF-derived exosomes in all groups, following the manufacturer’s instructions.

### 2.8 Bioinformatics and Statistical Analyses

Volcano plot was conducted to present the differentially expressed proteins based on the screening criteria. Hierarchical cluster was performed to find the same cluster of proteins. GO annotation was performed to discover the gene regulatory networks based on hierarchical categories according to the molecular function, biological process, and cellular component terms. Pathway analysis was performed with the Kyoto Encyclopedia of Genes and Genomes (KEGG) (http://www.genome.jp/kegg/ and https://david.ncifcrf.gov/) to characterize the enriched pathways. SPSS 20.0 (IBM, Armonk, NY) was used for statistical analysis. Quantitative variables were presented as mean ± standard deviation (SD) or the median and interquartile range; categorical variables were indicated as percentages (%). Comparisons of the differences of continuous variables were performed by one-way analysis of variance (ANOVA) or nonparametric tests. Categorical variables were compared with the chi-square test. A *p*-value < 0.05 was accepted as significant.

## 3 Results

### 3.1 Basic Characteristics of the Participants

The age of axSpA and gout was younger than OA. The serum uric acid (sUA) level of gout was higher than OA. The sUA levels of axSpA and RA were lower than OA. The C-reactive protein (CRP) and erythrocyte sedimentation rate (ESR) levels of gout were higher than OA. The rheumatoid factor (RF) and anti-cyclic citrullinated peptide antibody (anti-CCP) levels of RA were higher than OA. All the differences were significant (*p* < 0.05) (**Table 1**).

**Table 1:** Basic characteristics of the participants.

	Gout (*n* = 42)	axSpA (*n* = 10)	OA (*n* = 18)	RA (*n* = 30)	*p*-value
**Age (years)**	53.42 ± 12.60	29.00 ± 12.54*	61.72 ± 12.10	56.07 ± 10.13	<0.01
**Gender (male/female)**	36/6	8/2	6/12*	8/22*	<0.01
**Disease duration (years)**	7.39 ± 5.58	2.05 ± 3.10*	5.74 ± 7.82	8.36 ± 7.84	<0.05
**Disease status (active), *n* (%)**	42 (100.0%)	10 (100.0%)	18 (100.0%)	30 (100.0%)	–
**sUA (μmol/L)**	515.33 ± 148.97	295.40 ± 103.00*	350.29 ± 81.80*	326.58 ± 80.93*	<0.01
**CRP (mg/L)**	45.24 ± 42.09	60.46 ± 69.54*	12.91 ± 20.51*	45.49 ± 41.64	<0.01
**ESR (mm/h)**	75.99 ± 81.61	52.17 ± 43.10	33.13 ± 28.14*	77.96 ± 41.30	0.016
**RF (IU/ml)**	3.41 ± 3.70	2.40 ± 1.42	2.92 ± 2.27	123.57 ± 105.08*	<0.01
**Anti-CCP (U/ml)**	11.69 ± 5.56	12.76 ± 13.07	13.27 ± 4.08	69.79 ± 57.66*	<0.01
**Ongoing treatments**					
**Glucocorticoid, *n* (%)**	17 (40.5%)	3 (30.0%)	2 (11.1%)	10 (33.3%)	
**NSAIDs, *n* (%)**	36 (85.7%)	10 (100.0%)	18 (100.0%)	27 (90.0%)	
**Colchicine, *n* (%)**	17 (40.5%)	–	–	–	
**Uric acid lowering, *n* (%)**	23 (54.8%)	–	–	–	
**DMARDs, *n* (%)**	–	5 (50.0%)	–	24 (80.0%)	
**Anti-TNF-α, *n* (%)**	–	2 (20.0%)	–	1 (0.3%)	

*p < 0.05 vs. gout group.

### 3.2 The Isolation and Identification of SF-Derived Exosomes

**Figure 1:**
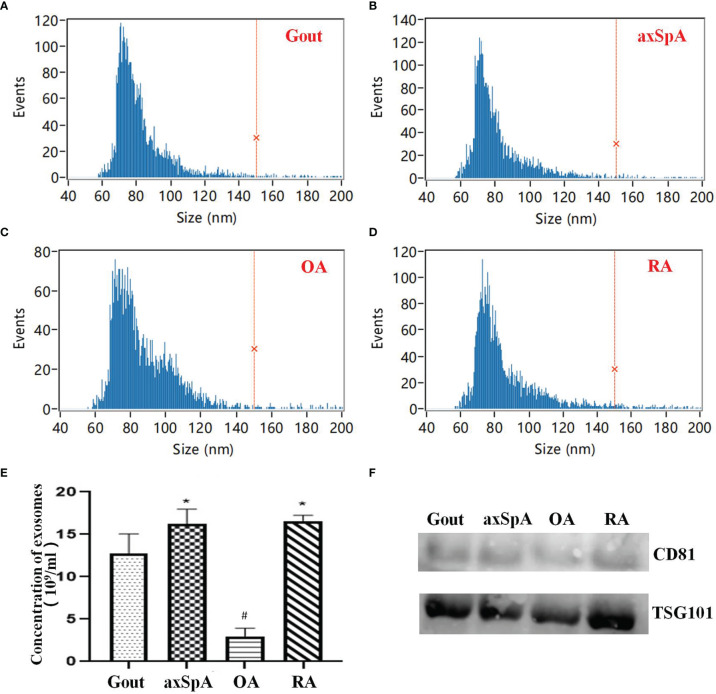
The isolation and identification of SF-derived exosomes. **(A–D)** The size of exosomes detected by HSFC for nanoparticle analysis (**A**: Gout; **B**: axSpA; **C**: OA; **D**: RA). **(E)** The comparison of exosome concentrations in the four groups based on HSFC for nanoparticle analysis. **(F)** The expression of CD81 and TSG101 was detected by Western blot. *p-value < 0.05 vs. gout group, ^#^ *p*-value < 0.05 vs. other groups.

HSFC for nanoparticle analysis results showed that the diameters of exosomes mainly ranged from 60 to 120 nm in all groups (**Figures 1A–D**). The concentrations of exosomes in gout, axSpA, RA, and OA were (12.70 ± 2.36), (16.23 ± 1.74), (16.60 ± 0.65), and (3.00 ± 0.95) ×10^9^ particles/ml. The concentrations of exosomes in axSpA and RA were higher than gout (*p* < 0.05). The concentrations of exosomes in OA was the lowest (*p* < 0.05) (**Figure 1E**). Western blot results indicated the expressions of CD81 and TSG101 in SF-derived exosomes (**Figure 1F**).

### 3.3 Quality Control of Proteomics Data

**Figure 2:**
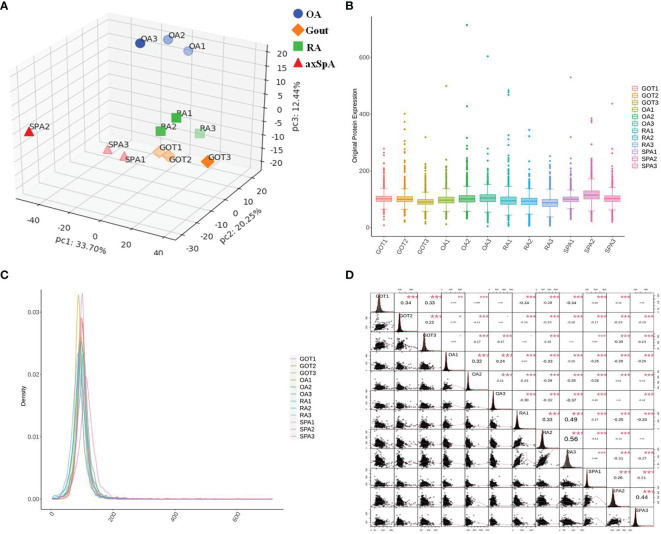
Quality control of proteomics data. **(A)** PCA analysis. **(B)** Box plot analysis. **(C)** Density map analysis. **(D)** Corrplot analysis. *p < 0.05, **p < 0.01, ***p < 0.001.

Principal component analysis (PCA) was used to indicate the the relationship between samples from different dimensions, and the result showed that protein expression of the samples in the same group were close (**Figure 2A**). Box plot analysis and density map analysis showed that the fluctuation of data in every sample was concentrated (**Figures 2B, C**). Corrplot analysis revealed that the correlation of the samples in the same group was stronger (**Figure 2D**).

### 3.4 Screening of Differentially Expressed Proteins

#### 3.4.1 Overall Distribution of Differentially Expressed Proteins

**Figure 3:**
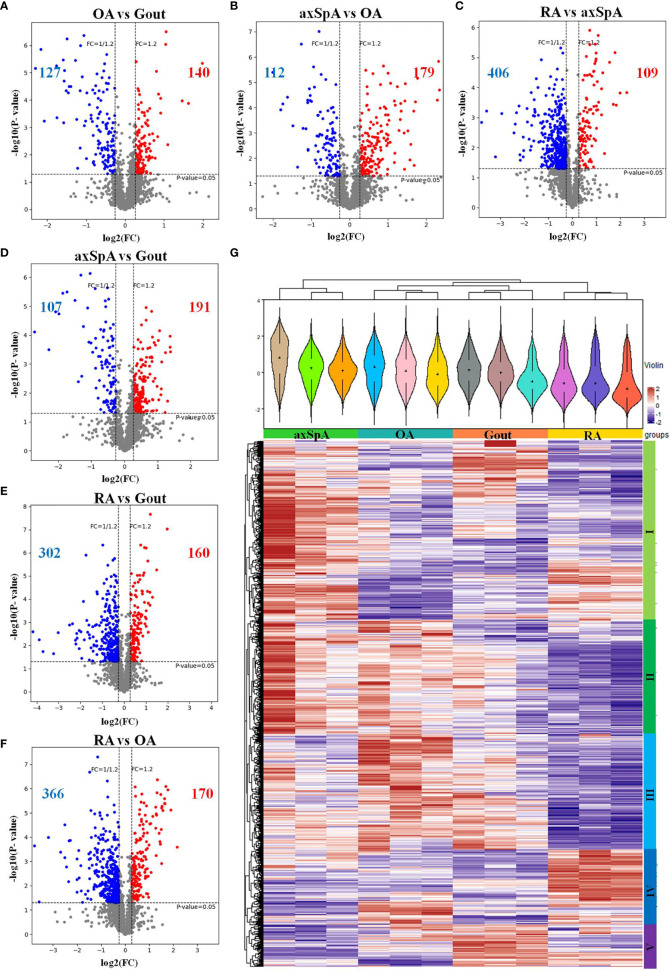
Overall distribution of differentially expressed proteins. **(A–F)** The differentially expressed proteins analyzed by volcano plots between every two groups (**A**: OA vs. Gout; **B**: axSpA vs. OA; **C**: RA vs. axSpA; **D**: axSpA vs. Gout; **E**: RA vs. Gout; **F**: RA vs. OA). **(G)** Hierarchical clustering analysis of the differentially expressed proteins in the four groups. In the color bar, red represents high expression, and purple represents low expression.

The differentially expressed proteins between every two groups were selected, according to the criteria of (log2 |fold-change| ≥ 1.2 and *p* < 0.05). The results showed that 140 proteins were found upregulated, while 127 were proteins downregulated in OA, compared with gout (**Figure 3A**). One hundred and seventy-nine proteins were found upregulated, while 112 proteins were downregulated in axSpA, compared with OA (**Figure 3B**). One hundred and nine proteins were found upregulated, while 406 proteins were downregulated in RA, compared with axSpA (**Figure 3C**). One hundred and ninety-one proteins were found upregulated, while 107 proteins were downregulated in axSpA, compared with gout (**Figure 3D**). One hundred and sixty proteins were found upregulated, while 302 proteins were downregulated in RA, compared with gout (**Figure 3E**). One hundred and seventy proteins were found upregulated, while 366 proteins were downregulated in RA, compared with OA (**Figure 3F**). Hierarchical clustering analysis was performed to reveal the dynamic profiles of differentially expressed proteins in the four groups. Accordingly, five clusters were identified, of which cluster I was the proteins mainly upregulated in axSpA, cluster II was the proteins mainly upregulated in axSpA and OA, cluster III was the proteins mainly upregulated in OA and gout, cluster IV was the proteins mainly upregulated in RA, and cluster V was the proteins mainly upregulated in gout (**Figure 3G**).

#### 3.4.2 Proteins Highly Expressed in Gout

**Figure 4:**
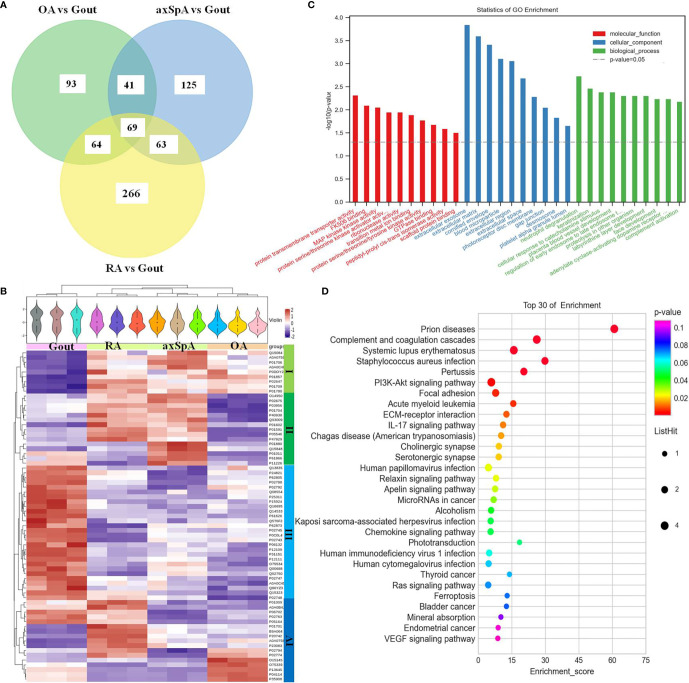
The screening and function analysis of proteins highly expressed in gout. **(A)** Venn diagram analysis of OA vs. Gout, axSpA vs. Gout, and RA vs. Gout. **(B)** Hierarchical clustering analysis of the differentially expressed proteins. **(C)** GO analysis of the highly expressed proteins. **(D)** KEGG pathway analysis of the highly expressed proteins.

Sixty-nine differentially expressed proteins were found in gout, compared with OA, axSpA, and RA simultaneously (**Figure 4A**). Hierarchical clustering analysis showed that four clusters were identified, of which cluster I was the lowly expressed proteins in gout and cluster III was the highly expressed proteins in gout (**Figure 4B**). With the criteria of unique peptides ≥ 2, 25 proteins were found highly expressed in gout uniquely, lysozyme C, alpha-1-acid glycoprotein 1, lactotransferrin, protein S100-A9, and myeloperoxidase included (**Table 2**). GO analysis showed that molecular function of highly expressed proteins were significantly enriched in “protein transmembrane transporter activity” (*p* = 0.005) and “FK506 binding” (*p* = 0.008). Regarding the biological progress, the proteins mediated “neutrophil degranulation” (*p* = 0.002) and “keratinization” (*p* = 0.003). GO analysis of cellular components indicated that most proteins were annotated as extracellular exosomes (**Figure 4C**). KEGG pathway analysis demonstrated that the proteins were significantly enriched in “prion diseases” (*p* = 3.91E-07) and “complement and coagulation cascades” (*p* = 1.21E-05) (**Figure 4D**).

**Table 2:** Proteins highly expressed in gout.

Name	Abundances											
	Gout			axSpA			OA			RA		
**LYZ**	227.3	217.4	222.4	72.5	62.7	63.8	51.5	48.7	35.1	68.9	63	66.6
**ORM1**	159.9	170.1	189.5	29.3	24.2	25.4	81.3	71.8	66.5	135.8	113.5	132.6
**LTF**	164.1	169.4	175.3	47.9	47.5	44.6	99.1	93.4	92.3	91.4	86.8	88.3
**S100A9**	165.6	152.4	153.5	35.9	33.8	41.5	78	80.5	79.8	123	129.2	126.9
**MPO**	149.5	153.2	150.2	60.6	62.5	57.9	73.5	71.6	71.8	115.8	118	115.4
**COL6A3**	146.1	146.4	149.4	99.9	101.5	96.8	73.5	63.5	69.1	80.7	84.8	88.3
**GNB1**	170.8	131.6	137.6	106.8	94.5	105.5	99.8	100.2	110.1	48.7	49.1	45.2
**H3-4**	145.8	154.5	133.9	71	78.8	72.7	84.9	82	102.1	88.4	99.1	86.8
**C9**	139.3	137.6	149.4	102	87.9	93.7	51.2	47.3	45.6	120.3	109.8	116
**FKBP3**	139.6	153.5	132.5	107	106.1	103.8	77.3	78	75.9	75.5	81.9	68.9
**MAP2K1**	147.5	144.9	127.9	96.1	110.5	106.8	82.9	88.6	89.7	64.8	75.9	64.3
**CSDE1**	150.9	141.2	127.9	108.8	117.9	112.6	63.9	62.4	76.9	81.6	83.6	72.1
**S100A7**	140.6	140.7	134.2	90.6	103.2	89.4	79	78.1	85.6	82.6	93.4	82.8
**H4C1**	136.4	138.3	138.8	65.3	67.4	69.1	94.6	95.6	91.2	101.5	101.6	100.2
**C4A**	135.5	137.3	139	99.4	97.5	97.2	96.1	95.4	93.6	68.8	70.5	69.7
**C1QA**	135.2	134.5	140.6	101.2	93.3	86.9	97.6	94.7	83.8	79.4	70	82.9
**TNC**	138.4	136.2	131.9	60.7	66.3	60.4	93.9	90.1	103.1	103.7	113.9	101.3
**HRNR**	116.6	132.4	150.9	107.4	90.2	89.4	86.7	83.2	66.5	95.1	87.1	94.3
**UBAP2**	144.9	132	120.9	91.2	76.2	91.4	97.2	87.7	95.1	93.3	74.1	96
**C1QC**	122.8	132.6	133.7	101.4	87.8	91.9	81.9	78.3	76.3	100.5	100	92.9
**FTL**	136.9	126	122.5	70.1	72.4	80.4	107.2	101.3	112	90	93.5	87.7
**APCS**	124.5	127	129.1	104.1	96.2	96.7	104.4	98.1	92.5	76.1	74.9	76.4
**DSC1**	124.8	123.1	118.7	87.1	102.2	87	101.8	97	89	91.7	91.6	86.1
**DSP**	119.8	122.1	111.5	89.9	94.3	96.5	91.5	94.7	97.4	91.2	100.5	90.8
**AZGP1**	118.9	118.1	116	94	92.9	90.7	99.6	97.6	89.9	94.2	93.2	94.9

#### 3.4.3 Proteins Highly Expressed in axSpA

**Figure 5:**
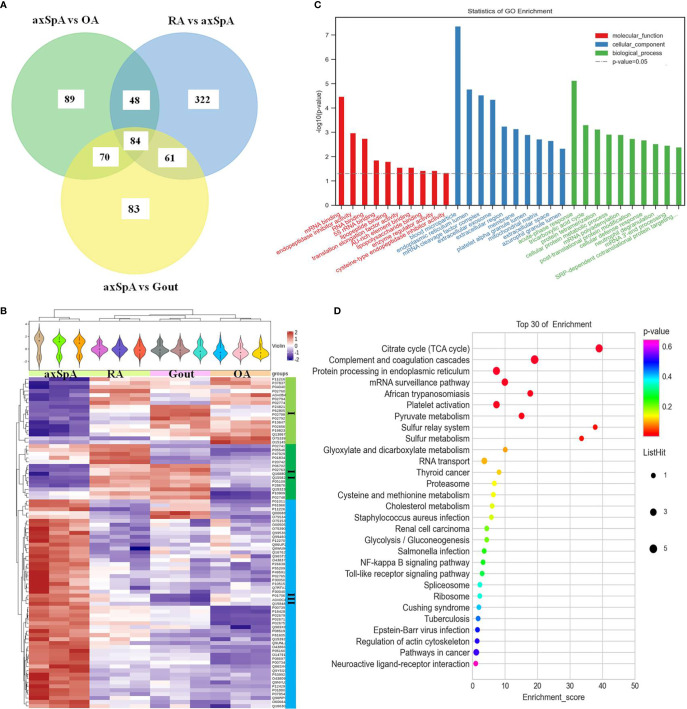
The screening and function analysis of proteins highly expressed in axSpA. **(A)** Venn diagram analysis of axSpA vs. OA, axSpA vs. Gout, and RA vs. axSpA. **(B)** Hierarchical clustering analysis of the differentially expressed proteins. **(C)** GO analysis of the highly expressed proteins. **(D)** KEGG pathway analysis of the highly expressed proteins.

Eighty-four differentially expressed proteins were found in axSpA, compared with OA, gout, and RA simultaneously (**Figure 5A**). Hierarchical clustering analysis showed that three clusters were identified, of which cluster I was the lowly expressed proteins in axSpA and cluster III was the highly expressed proteins in axSpA (**Figure 5B**). With the criteria of unique peptides ≥ 2, 39 proteins were found highly expressed in axSpA uniquely, RNA-binding protein 8A, alpha-2-HS-glycoprotein, protein transport protein Sec24C, alpha-2-antiplasmin, and translocon-associated protein subunit gamma included (**Table 3**). GO analysis showed that the molecular function of highly expressed proteins was significantly enriched in “mRNA binding” (*p* = 3.33E-05) and “endopeptidase inhibitor activity” (*p* = 0.001). Regarding the biological progress, the proteins mediated “acute-phase response” (*p* = 7.34E-06) and “tricarboxylic acid cycle” (*p* = 0.0005). GO analysis of the cellular components indicated that most proteins were annotated as blood microparticle (**Figure 5C**). KEGG pathway analysis demonstrated that the proteins were significantly enriched in “citrate cycle (TCA cycle)” (*p* = 2.74E-06) and “complement and coagulation cascades” (*p* = 7.10E-05) (**Figure 5D**).

**Table 3:** Proteins highly expressed in axSpA.

Name	Abundances											
	Gout			axSpA			OA			RA		
**IGHD**	82	79.3	70.2	183.4	215.7	199.5	60.3	61.5	66.2	57.9	67	57.1
**RBM8A**	67.1	82.4	74.5	207.2	187.3	188.2	56.3	54.6	66.9	75.1	63.1	77.2
**AHSG**	79.2	86	56.2	151.5	234.3	157.6	51.7	62.5	69.2	67.1	104.8	79.8
**SEC24C**	73	77	72.7	165.7	193.3	166.3	64.9	85.4	65.4	72	75.4	88.7
**FGG**	89.1	90.1	95.6	171	178.8	159.9	35.9	35.4	29.9	105.6	103.5	105.1
**FGA**	93.8	94	95.5	161.1	170	169	35	33.6	31.8	103.9	109.1	103.2
**DLAT**	87.3	77.9	66.6	120.5	187.1	166.5	74.6	101.9	62.9	86.8	97.7	70.1
**FGB**	85.2	90.1	92.9	170.3	149.3	153.4	34.8	34.3	28	122.9	116.6	122.2
**SERPINF2**	87.5	94	92.5	164.9	148.1	153.6	60.7	58.6	53.5	95	94.1	97.6
**APOL1**	94	92.5	93.1	161	144.7	149	63.6	60.8	56.4	95.9	92.9	96.2
**SSR3**	73.8	68.2	72.2	148.8	141.6	156.3	80.1	78.3	81.5	113.2	108.7	77.3
**NUDT21**	82.1	87.2	78.4	146.1	159.1	136.2	76.9	83.3	86.9	93.5	78.1	92
**IDH3B**	99	111.8	65	135.7	155.1	149	76.6	101	74.5	70.5	91.7	70.1
**LBP**	97.7	93.1	104.2	139.8	148.2	137.4	51	53.2	51.9	103.5	112.2	107.8
**F2**	98.7	99.3	100.2	144	137.2	143.2	70	68	64.3	92.3	92.3	90.5
**DCD**	76.2	74	70.7	131.1	148.2	142.1	72.6	75.1	73.6	104.6	121.7	110
**TPR**	86.4	95.4	77.3	132.3	153	135.4	83.6	96.7	108.8	73.9	83.2	73.9
**FH**	95.7	93.4	82.5	133	147.6	138.6	84.9	89.9	81	89	86.7	77.6
**CD5L**	89.1	89	83.6	138.8	134.4	139.2	74.9	75.1	74.3	96.5	105.4	99.6
**VAT1**	94.1	94.4	89	142.1	145	122.4	83.2	85.5	99.8	77.7	85.9	80.9
**SERPINA3**	116	112	110.5	126	141.8	141.1	68.2	68.3	68.5	79.6	86.5	81.6
**HPR**	94.5	94.7	98.7	142.1	137.1	128.5	62.9	59.9	58.6	107.4	109.9	105.6
**ANXA3**	98.4	94.3	82.8	130.1	150.4	125.7	80.9	81.8	81.9	93.5	91.6	88.5
**SARS1**	92.2	90.8	78.8	117.7	155.2	129.3	72.7	82.3	86.6	101	102.2	91.1
**KTN1**	88.3	85.1	98.8	129.8	130.9	138.7	99.3	88.4	116.1	80.4	75.3	68.9
**GFM1**	84.6	96.1	85.7	125.9	142.7	127.3	79.6	89.3	84.5	103.8	80.9	99.6
**LPA**	92	89.2	80.6	120.8	141.1	128.5	72.8	78.5	83.7	106.1	101.2	105.4
**UGGT1**	101.8	94.2	85.6	124.3	133	128.6	87.2	87.4	77	97.4	92	91.6
**CS**	96	97.5	95.1	112	147.7	125.3	91.9	81.5	99.3	83.7	94.9	75
**RPL12**	98.7	97.4	92.1	110.4	145.8	122.2	76.7	83.2	92.4	94.2	100.4	86.6
**CPSF6**	83	104.2	79.5	130.4	116.6	124	96.7	89.3	69.7	96.9	107	102.8
**PSMD1**	93.7	94	86.5	117.7	129.6	122.5	94.6	98.4	106.9	90.4	85.3	80.4
**ZC3H15**	104.9	96.1	93.8	116.2	129	121.7	97.7	98.4	101.5	92.3	82.4	66
**TST**	108.5	92.1	98.2	115.3	127.4	124	93.5	88.2	104.4	85.7	84.4	78.4
**ERP29**	97.7	92.9	79.4	109.2	137.8	116.3	94.7	95.9	91.3	100.1	94.4	90.4
**KPNA3**	104.5	94.9	80.2	112.9	126.7	123.2	89.2	93.3	109	81.4	99.5	85.2
**TARS1**	96.9	97.3	89.4	118.6	124.9	116.4	90.3	83.6	97.8	99.4	98	87.4
**CLUH**	101.1	99.1	80.9	114.5	125.5	118.6	85.4	88	109.3	94.4	94.8	88.5
**NAP1L1**	98.9	102.8	87.8	113.2	123.1	116.1	89.8	85.5	90.6	101.8	101.5	89.1

#### 3.4.4 Proteins Highly Expressed in RA

**Figure 6:**
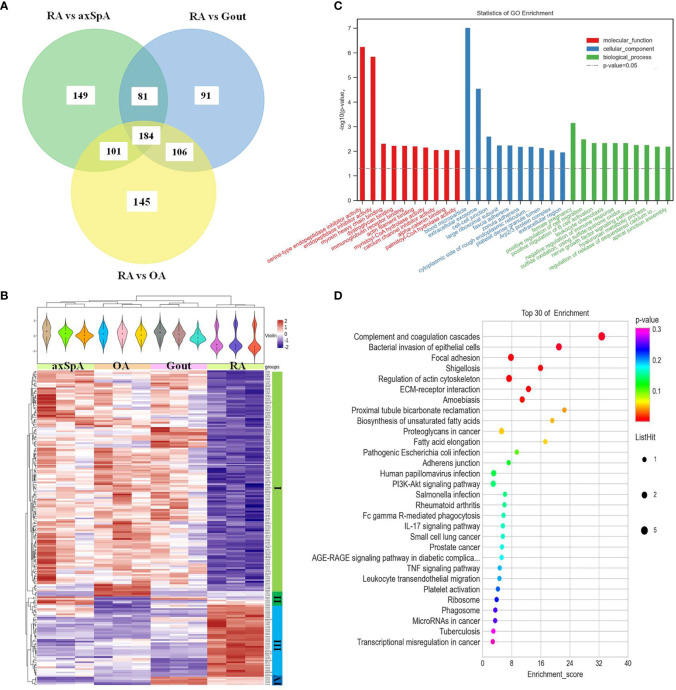
The screening and function analysis of proteins highly expressed in RA. **(A)** Venn diagram analysis of RA vs. OA, RA vs. Gout, and RA vs. axSpA. **(B)** Hierarchical clustering analysis of the differentially expressed proteins. **(C)** GO analysis the highly expressed proteins. **(D)** KEGG pathway analysis of the highly expressed proteins.

One hundred and eighty-four differentially expressed proteins were found in RA, compared with OA, gout, and axSpA simultaneously (**Figure 6A**). Hierarchical clustering analysis showed that four clusters were identified, of which cluster I was the lowly expressed proteins in RA and cluster III was the highly expressed proteins in RA (**Figure 6B**). With the criteria of unique peptides ≥ 2, 28 proteins were found highly expressed in RA uniquely, pregnancy zone protein, stromelysin-1, coronin-1A, vimentin, and heparin cofactor 2 included (**Table 4**). GO analysis showed that the molecular function of highly expressed proteins were significantly enriched in “serine-type endopeptidase inhibitor activity” (*p* = 5.59E-07) and “endopeptidase inhibitor activity” (*p* = 1.39E-06). Regarding the biological progress, the proteins mediated “female pregnancy” (*p* = 0.00069) and “positive regulation of B cell activation” (*p* = 0.00449). GO analysis of the cellular components indicated that most proteins were annotated as blood microparticle (**Figure 6C**). KEGG pathway analysis demonstrated that the proteins were significantly enriched in “complement and coagulation cascades” (*p* = 2.51E-07) and “bacterial invasion of epithelial cells” (*p* = 0.0003) (**Figure 6D**).

**Table 4:** Proteins highly expressed in RA.

Name	Abundances											
	Gout			axSpA			OA			RA		
**PZP**	49.9	46.8	46.6	103.1	91.9	95	70.9	69	62.5	187.2	186.8	190.4
**MMP3**	84.7	83.6	82	83.8	84.4	83.6	53.8	54.9	56.6	174.3	181.7	176.6
**ITIH4**	83.3	84.2	91.6	86.7	74.9	83.3	64.2	62	59.9	179.4	153.9	176.5
**CORO1A**	90	86.4	94.1	70.3	63.2	84.4	64.2	69.3	69.8	167.2	169.2	172
**VIM**	79.6	98.7	69.8	86.3	79.7	84.2	62.1	70.4	71	165.5	170.4	162.3
**IGHA2**	77	73.7	75.5	83.9	70.3	72.4	90.9	89.5	84.1	169.2	149.3	164.3
**CRP**	64.7	65.6	66.3	133.7	132.8	105.7	60.9	57	61.5	151.5	150.1	150.1
**KNG1**	89.1	91.2	98.5	86.1	74.5	81.4	79.4	73.7	75.2	156.2	139.3	155.4
**IGHG4**	100.5	96.6	86.9	68.5	80.7	73	78.2	78.9	91.3	137.6	168.7	139.1
**IGLV1-51**	65.3	67.8	60.8	94.9	101	90.8	90	91.4	96.9	142.8	158.5	139.8
**SERPIND1**	84.8	78.6	82.7	125.6	117.5	117.7	51.5	49.7	51.7	147.3	141.5	151.4
**VTN**	101.3	103.7	106.4	116.7	98.2	106	43.5	44	41.3	148.8	142.4	147.8
**IGHA1**	95.4	95.6	101.6	83.9	76.2	83.9	80.6	79.3	73.6	143.3	140	146.6
**IGHV3-49**	76.8	80.9	76.4	97.8	98.1	88.1	86.2	80.6	88.9	145.2	139.2	141.7
**FN1**	86.7	88.6	90.3	91.2	87	84.2	90.7	89.8	87.9	134.9	132.3	136.6
**IGKC**	74.1	74	80.8	102.3	98.6	125	83.8	80.5	78.6	135.5	130.2	136.7
**CCAR1**	68.2	77.5	77	73.8	102.2	100.7	100	94.4	105.1	151.5	130.2	119.4
**ARPC4**	93.6	94.4	85.7	89	93.7	93.2	85.6	80.2	90.9	125.8	144.8	123.1
**SLC25A10**	70	77.5	74.7	96.8	97.4	76.5	113.7	105.5	96.3	139.9	128.5	123.2
**RPL27**	107.5	109.4	92.5	82.5	97.6	85.4	74.5	78.4	81.4	127.6	142.5	120.7
**IGLL5**	76.1	78.1	75.8	105.7	100	90.1	96.9	94.7	95.7	129.9	128.7	128.3
**SERPINA1**	109.6	97.8	104.3	91.1	76.3	82.3	85.6	80.2	88.1	131.9	125.6	127.3
**CLIC1**	78.4	86.9	84.6	97.8	98.7	100.1	91.5	90.4	92.6	134.6	117.8	126.6
**AMBP**	100	103.1	102.7	77.2	76.2	73.5	101.5	100.2	92.2	124.9	122.9	125.6
**IGLV3-10**	77.5	79.2	79.4	103.6	93.2	95.1	106.3	104.2	93.6	126	120.2	121.7
**FGB**	85.2	90.1	92.9	170.3	149.3	153.4	34.8	34.3	28	122.9	116.6	122.2
**ACOT7**	86	90	81.4	98.1	109.8	87.6	100.8	95.5	90.3	120.9	118.6	121
**VCL**	90.5	87.6	87.1	101.1	94.1	104	88.1	96.7	90.3	114.6	118.9	127

### 3.5 Verification of Exosome-Derived PZP in SF by ELISA

**Figure 7:**
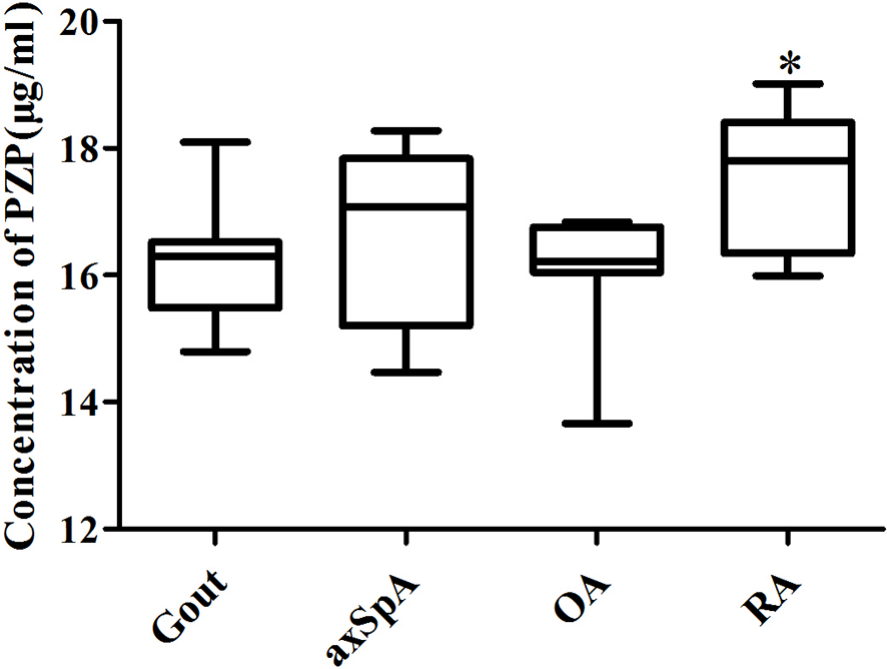
Verification of exosome-derived PZP in SF by ELISA. The ELISA kits were performed to detect the PZP level of SF-derived exosomes in gout, axSpA, OA, and RA. *P value < 0.05 vs. OA groups.

ELISA results showed that exosome-derived PZP levels of SF in gout, axSpA, OA, and RA were 16.29 (15.48, 16.52), 17.08 (15.20, 17.83), 16.21 (15.99, 16.76), and 17.79 (16.34, 18.41) μg/ml. The level of exosome-derived PZP in RA was higher than OA (*p* < 0.05) (**Figure 7**). It showed consistency with the TMT-LC-MS/MS data.

## 4 Discussion

Exosomes in SF have a close relationship with the pathogenesis of arthritis, whose biological contents have been used as potential biomarkers. In our study, the proteomics of SF-derived exosomes in RA, axSpA, gout, and OA were explored, and the results showed that the protein profiles of SF-derived exosomes were different. The function analysis indicated that the highly expressed proteins in IA may potentially participate in immune response.

IA is characterized by synovial inflammation and synovial hyperplasia, with the change of volume and composition in SF (14). SF in IA patients contains abundant inflammatory cytokines and immune cells, playing an important role in the molecular mechanisms of IA (7). Analyzing the composition of SF is helpful to clarify the pathogenesis and distinguishing biomarkers of IA. Recently, the function of exosomes in SF was established (15, 16). Exosomes in SF can lead to inflammation, degeneration of cartilage, and destruction of joints (10). It has been reported that SF-derived exosomes of RA patients may contain the disease-specific “synovial signature of osteoclasto-genesis” (11). Foers (13) indicated that 45 and 135 EV-associated proteins were significantly elevated in RA with high-level inflammation than in RA with low-level inflammation and OA, respectively. However, no study has compared the proteomics of SF-derived exosomes in RA, axSpA, gout, and OA simultaneously.

In our study, SF-derived exosomes were isolated from RA, axSpA, gout, and OA patients. The TMT-labeled quantitative proteomics technique was used to explore the protein profiles of exosomes. The differentially expressed proteins between every two groups were selected, according to the criteria of (log2 |fold-change| ≥ 1.2 and *p* < 0.05). The uniquely expressed proteins were further screened, based on the intersection of differentially expressed proteins between every two groups. The result showed that 25 proteins were found highly expressed in gout uniquely, lysozyme C, alpha-1-acid glycoprotein 1, and protein S100-A9 included. Thirty-nine proteins were found highly expressed in axSpA uniquely, RNA-binding protein 8A and protein transport protein Sec24C included. Twenty-eight proteins were found highly expressed in RA uniquely, pregnancy zone protein and stromelysin-1 included. Twenty-eight proteins were found highly expressed in OA uniquely, cartilage intermediate layer protein 1 and stromal interaction molecule 1 included (**Supplementary Table 1**). Highly expressed proteins in exosomes have a close relationship with their functions, which may be the potential biomarkers (13). It is the first time to indicate the highly expressed proteins in SF-derived exosomes of RA, axSpA, gout, and OA. It may lay the foundation for the study of molecular mechanisms and biomarkers for IA.

Lysozyme C in SF-derived exosomes of gout was higher than other groups, while Lysozyme C in RA and axSpA was higher than that in OA. It means that Lysozyme C may have a close relationship with IA. Lysozyme is an antimicrobial enzyme, which is found in monocytes and macrophages, forming part of the innate immune system (17, 18). Bennett (19) compared the lysozyme levels of SF in patients with traumatic effusions, OA, RA, pseudogout, septic arthritis, and gout, and the results showed that elevated lysozyme levels were found in all the IA. Klockars (20) indicated that patients with rheumatoid disease had significantly higher levels of lysozyme in synovial fluid than patients with non-rheumatic diseases. It is consistent with our study. In addition, protein transport protein Sec24C in SF-derived exosomes of axSpA was higher than other groups, while Sec24C in RA, gout, and OA were close. It suggests that Sec24C may be the specific protein of axSpA. Sec24C is an essential coat protein II (COPII) component, which is involved in protein transports from the endoplasmic reticulum (ER) (21, 22). It is worthy to pay more attention to the function of Sec24C in axSpA. Besides, PZP in SF-derived exosomes of RA was higher than other groups, while PZP in axSpA and OA was higher than gout, and PZP in axSpA was higher than OA. The ELISA results indicated that the level of exosome-derived PZP in RA was higher than that in OA, gout, and axSpA, showing consistency with the TMT-LC-MS/MS data. PZP is a high-molecular-weight glycoprotein that is initially described as elevated in the serum of women during pregnancy. Finch found that PZP was released into neutrophil extracellular traps (NETs) and reported a novel link between airway infection (23). It had been reported that PZP of serum-derived exosomes in inflammatory bowel disease was higher than healthy people (24). Skornicka confirmed that PZP could selectively modulate T-cell activation (25). That is to say, PZP may participate in the occurrence and development of inflammation.

As for the bioinformatic analysis of the highly expressed proteins in every group, the highly expressed proteins in gout including alpha-1-acid glycoprotein 1, desmocollin-1, and hornerin were significantly involved in “neutrophil degranulation”. Neutrophils are key effector cells of innate immunity, which can be triggered by monosodium urate (MSU) crystals, resulting in the acute symptoms of gout (26). Neutrophils may kill pathogens by degranulation and the release of NETs extracellularly. It has been reported that the production of NETs embedding MSU crystals was a possible mechanism of the inflammatory phase during gout (27). It is worthy to explore the relationship between neutrophil degranulation and gout as a next step. The highly expressed proteins in axSpA including citrate synthase, fumarate hydratase, isocitrate dehydrogenase [NAD] subunit beta, and the dihydrolipoyllysine-residue acetyltransferase component of pyruvate dehydrogenase complex were significantly involved in “citrate cycle (TCA cycle)”. The citrate cycle is an important aerobic pathway for the final steps of the oxidation of carbohydrates and fatty acids. Recent evidence confers a new role of TCA cycle in immunity (28). Cytosolic metabolism of citrate to acetyl-coenzyme A (acetyl-CoA) is important for both fatty-acid synthesis and protein acetylation, both of which have been linked to macrophage and dendritic cell activation (29). As for the relationship between citrate cycle and axSpA, it has not been reported yet. The highly expressed proteins in RA including vitronectin, heparin cofactor 2, fibrinogen beta chain, alpha-1-antitrypsin, and kininogen-1 were significantly involved in “complement and coagulation cascades”. Complement and coagulation is critical for effecting an appropriate innate response (30). Much more attention should be paid to the relationship of complement and coagulation and RA. The bioinformatic analysis of the highly expressed proteins in OA indicated that they are mainly involved in “negative regulation of neuron death” and “mRNA surveillance pathway”, which was less associated with inflammation (**Supplementary Figure 1**). The hypothetical molecular mechanisms proposesed above may provide therapeutic insights for IA.

There are some limitations in our study. Firstly, a small number of patients were included in the exploratory study. Secondly, further analyzing the significance of the highly expressed proteins is needed. Thirdly, the function of these proteins should be evaluated *in vitro* and *in vivo*.

In conclusion, the protein profiles of SF-derived exosomes in RA, axSpA, gout, and OA patients were different. Some potential biomarkers and hypothetical molecular mechanisms were proposesed, which may provide helpful diagnostic and therapeutic insights for IA.

## Data Availability Statement

The datasets presented in this study can be found in online repositories. The names of the repository/repositories and accession number(s) can be found at: http://proteomecentral.proteomexchange.org; PXD029448.

## Ethics Statement

The studies involving human participants were reviewed and approved by EC office of Guangdong Second Provincial General Hospital (2017-FSMY-009). The patients/participants provided their written informed consent to participate in this study.

## Author Contributions

YH, WD, and TL designed the study. YL, QH, SS, ZJ, LH, ZL, and XH conducted experiments and performed analyses. YH and YL drafted the manuscript. ZL and XH supervised the study. All authors approved the final manuscript as submitted and agreed to be accountable for all aspects of the work.

## Funding

This study was funded by Science and Technology Projects in Guangzhou, China (Nos. 202102020127 and 202102080321), and Doctoral Workstation Foundation of Guangdong Second Provincial General Hospital (No. 2019019).

## Conflict of Interest

The authors declare that the research was conducted in the absence of any commercial or financial relationships that could be construed as a potential conflict of interest.

## Publisher’s Note

All claims expressed in this article are solely those of the authors and do not necessarily represent those of their affiliated organizations, or those of the publisher, the editors and the reviewers. Any product that may be evaluated in this article, or claim that may be made by its manufacturer, is not guaranteed or endorsed by the publisher.
