# Supplementary figures and images for: TMT-Based Quantitative Proteomics Analysis of Synovial Fluid-Derived Exosomes in Inflammatory Arthritis
Source: Front Immunol. 2022 Mar 11;13:800902. doi: 10.3389/fimmu.2022.800902 (PMC8961740; doi:10.3389/fimmu.2022.800902)

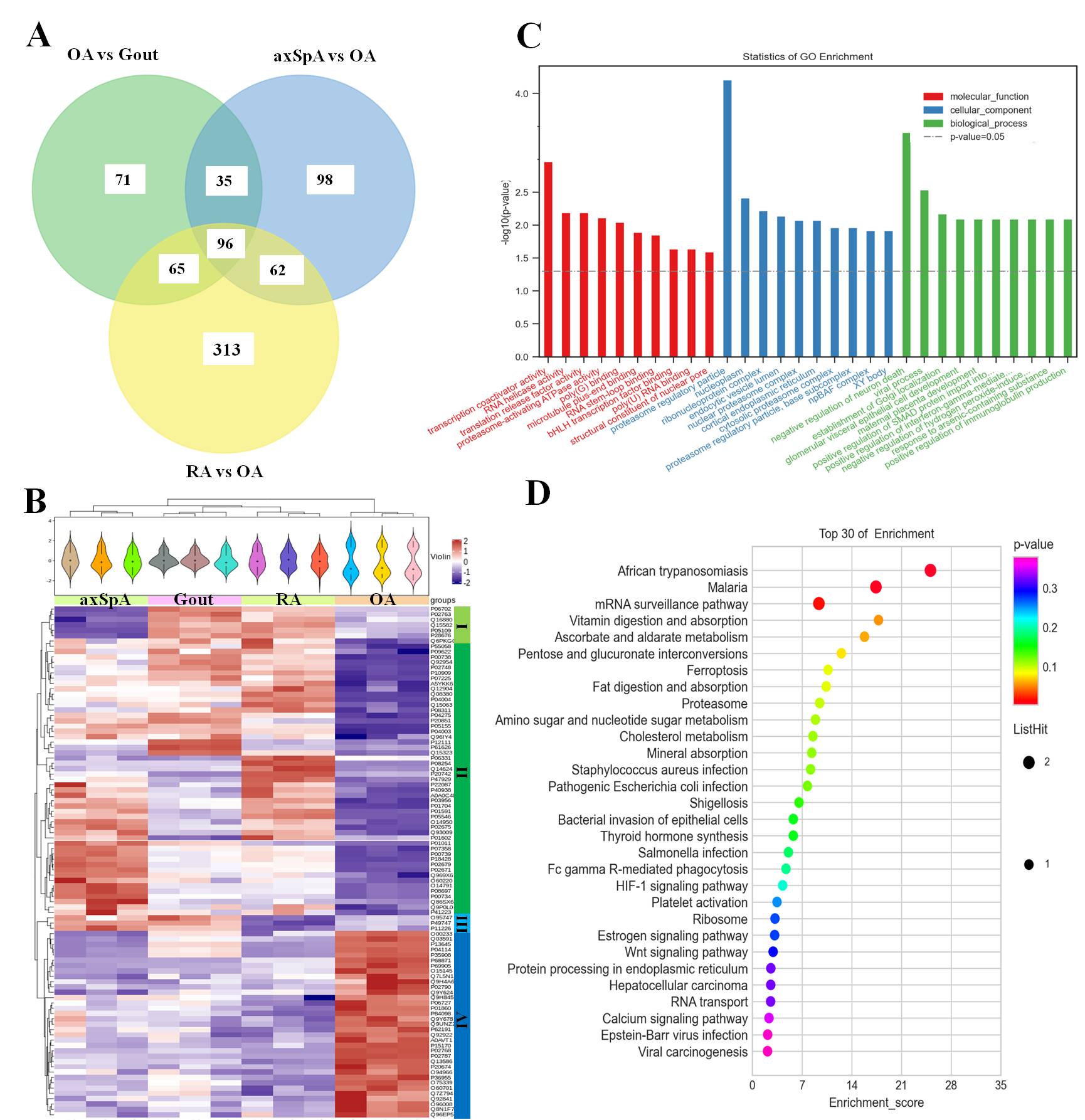

Supplement: Supplementary file 1 [file Image_1.jpeg]
