# Supplementary material for: TMT-Based Quantitative Proteomics Analysis of Synovial Fluid-Derived Exosomes in Inflammatory Arthritis
Source: Front Immunol. 2022 Mar 11;13:800902. doi: 10.3389/fimmu.2022.800902 (PMC8961740; doi:10.3389/fimmu.2022.800902)
Supplement: Supplementary file 2 [file Table_1.docx]

**Suppletment Table 1. Proteins highly expressed in OA group**

| **Name** | **Abundances** | | | | | | | | | | | |
| --- | --- | --- | --- | --- | --- | --- | --- | --- | --- | --- | --- | --- |
|  | **Gout** | | | **axSpA** | | | **OA** | | | **RA** | | |
| **HBB** | 55.1 | 52.1 | 51.1 | 52.3 | 52.8 | 51.1 | 216 | 210 | 200.7 | 85.2 | 86.6 | 86.9 |
| **HBA1** | 60.8 | 62.4 | 66.8 | 56.5 | 50.6 | 60.8 | 212 | 191.1 | 181.9 | 90.8 | 77.6 | 88.7 |
| **ARPC3** | 74.2 | 66.4 | 64.4 | 58.3 | 60.4 | 50.8 | 181.1 | 181.5 | 203 | 91.3 | 84 | 84.6 |
| **ALB** | 86.8 | 86.8 | 83.8 | 73.9 | 74.6 | 73.4 | 174.5 | 176.8 | 179.7 | 62.5 | 63.6 | 63.5 |
| **HPX** | 71 | 76.2 | 87.1 | 90.2 | 70 | 75.3 | 189.1 | 170.2 | 139.8 | 85.2 | 68.1 | 77.7 |
| **TF** | 78.7 | 80.3 | 77 | 87.9 | 84.7 | 81.7 | 164 | 161.6 | 164.3 | 73.4 | 73.3 | 73.1 |
| **CILP** | 100.2 | 107.8 | 98.9 | 79.6 | 84.9 | 75 | 164.7 | 153.1 | 158.6 | 58.6 | 60 | 58.6 |
| **STIM1** | 84.7 | 88.7 | 88 | 89.8 | 94.9 | 82.2 | 153.7 | 155.1 | 165.8 | 66.7 | 60.5 | 70.1 |
| **GSPT1** | 87.3 | 82.8 | 72.7 | 77.8 | 101.3 | 74.7 | 154.4 | 153 | 150.7 | 93.9 | 76.7 | 74.7 |
| **PSMC1** | 94.1 | 105.6 | 61.1 | 80.8 | 109.8 | 104.9 | 141.6 | 160.1 | 143.7 | 76 | 62.3 | 60 |
| **SERPINF1** | 101.9 | 101.8 | 85.4 | 82.5 | 80.8 | 87.9 | 143.6 | 157 | 143.7 | 70 | 77 | 68.5 |
| **KRT2** | 108.6 | 107.9 | 112.8 | 72.1 | 67.9 | 73.2 | 151.3 | 150 | 142.4 | 71.3 | 69.6 | 72.8 |
| **PSMD9** | 106.9 | 107.8 | 106 | 73.8 | 72.8 | 68.4 | 139 | 134.9 | 132.3 | 95.8 | 68.7 | 93.6 |
| **APOB** | 105.4 | 103.6 | 107.4 | 81.5 | 77.5 | 81 | 136 | 133 | 132.9 | 80.9 | 79 | 81.8 |
| **KRT10** | 108.9 | 109.1 | 110.8 | 77.8 | 76.2 | 76.1 | 133.4 | 134.4 | 133.4 | 78.8 | 79.5 | 81.6 |
| **RPL19** | 92.2 | 96 | 91.2 | 96.5 | 111.6 | 95.6 | 128.3 | 128.2 | 137.5 | 73.1 | 75.7 | 74.1 |
| **NSFL1C** | 92.5 | 88.3 | 80.1 | 92.8 | 111.3 | 96.8 | 134 | 127.4 | 127.7 | 82.5 | 89.2 | 77.4 |
| **DAZAP1** | 88.2 | 100.3 | 76.4 | 89.1 | 99.9 | 76.7 | 123.6 | 125.4 | 137.8 | 85.5 | 109.8 | 87.2 |
| **IGHG3** | 99.4 | 97.4 | 89.3 | 91 | 96.1 | 94.4 | 123.9 | 124.1 | 135 | 80.3 | 88.5 | 80.6 |
| **ACAD9** | 107.3 | 99.7 | 98 | 88.2 | 108.3 | 106.8 | 130.2 | 123.5 | 126 | 83.3 | 81.6 | 47.1 |
| **UGDH** | 101.7 | 104.4 | 92.4 | 94.9 | 93.5 | 89 | 132 | 122.2 | 123.7 | 90.9 | 75.1 | 80.1 |
| **NUP93** | 96.9 | 96.4 | 90.2 | 90.5 | 92.4 | 84.3 | 117.6 | 123.5 | 134.5 | 87.9 | 94.6 | 91.3 |
| **COPG1** | 94.1 | 94.2 | 82.8 | 90.5 | 114.5 | 94.6 | 132.6 | 117.7 | 123.7 | 85.1 | 92.9 | 77.2 |
| **COPS6** | 101.8 | 86 | 96.3 | 84.7 | 85.5 | 78.6 | 133.7 | 121.9 | 117.9 | 101.2 | 92.3 | 100.1 |
| **SMARCC1** | 103.7 | 93.6 | 79.9 | 91.1 | 108.2 | 94.2 | 118.9 | 121 | 124.5 | 93.5 | 81.9 | 89.6 |
| **DDX17** | 95.1 | 97.6 | 93.2 | 97.2 | 88.8 | 102 | 110.5 | 112 | 131.4 | 87.7 | 95 | 89.5 |
